# Pollution, Inflammation, and Vaccines: A Complex Crosstalk

**DOI:** 10.3390/ijerph18126330

**Published:** 2021-06-11

**Authors:** Laura Franza, Rossella Cianci

**Affiliations:** 1 Emergency Medicine, Catholic University of the Sacred Heart, Fondazione Policlinico Universitario A. Gemelli IRCCS, Largo A. Gemelli, 8-00168 Rome, Italy; cliodnaghfranza@gmail.com; 2 Dipartimento di Medicina e Chirurgia Traslazionale, Catholic University of the Sacred Heart, Fondazione Policlinico Universitario A. Gemelli IRCCS, Largo A. Gemelli, 8-00168 Rome, Italy

**Keywords:** pollution, vaccination, inflammation, immunity, microbiota

## Abstract

The importance of pollution in determining human health is becoming increasingly clear, also given the dramatic consequences it has had on recent geopolitical events. Yet, the consequences of contamination are not always straightforward. In this paper, we will discuss the effects of different pollutants on different aspects of human health, in particular on the immune system and inflammation. Different environmental pollutants can have different effects on the immune system, which can then promote complex pathologies, such as autoimmune disorders and cancer. The interaction with the microbiota also further helps to determine the consequences of contamination on wellbeing. The pollution can affect vaccination efficacy, given the widespread effects of vaccination on immunity. At the same time, some vaccinations also can exert protective effects against some forms of pollution.

## 1. The Interaction between Humans and the Environment, a Focus on Health

The importance of the environment on human health is a well-known fact. It is speculated that certain cultures would not have been born if it were not for the favorable environmental conditions. Modern technology allows most developed countries to modify the surrounding environment to their advantage. Those same societies are the ones which create the most pollution, a key factor in modifying the environment. Yet, the dramatic climate changes, for instance, have a massive toll, mostly on those who live in less developed countries.

Pollution is defined as an unfavorable alteration of the environment as a result of human activity. It is possible to classify pollution into three main classes—air, water, and land. Human activities can cause also other forms of pollution, such as noise and light pollution [1].

Alongside the more evident consequences of massive natural catastrophes, climate change and pollution have had a serious, yet less evident, impact on human health [2]. In this review, we will concentrate mostly on the three main forms of pollution, but it is worth noting that other forms of pollution also have an impact on human health, even though the underlying mechanisms are not always completely clear.

For instance, it has been observed that there is a significant relationship between light pollution, which is caused by anthropogenic and artificial electric light in the night environment, and cancer [3]. The cause of such apparently strange association of events, probably roots back to the important consequences of light pollution on metabolism, which in turn is caused by disruption of circadian rhythm, as seen, in particular, in murine models [4]. Interestingly, a conscious sleep disruption is not necessary to determine alterations of the circadian rhythm [5]. Similar results have been observed in humans, whose alteration of circadian rhythm is also linked to metabolic shifts and a tendency to lose muscle mass and gain weight [6,7].

The importance of air, water, and land pollutants on human health is undeniable and has been identified as one of the primal drivers in health inequality even in the developed world [8]. An example is the water crisis in Flint, which has led to serious consequences, particularly in children [9]. Contamination of food and drinking water is a consequence of water and land pollution, and typically affects the most delicate parts of the population. The effects are often mediated by the gut microbiota and intestinal niche immunity, and range from neurological disorders to cancer, and endocrine disruption [10]. The underlying mechanisms and the long-term consequences are not yet completely clear; thus, the effects might be even more serious than expected.

Overall, the effects of pollution on human health are complex and vast. In this narrative review, we will concentrate on the effects of pollution on the immune system and will then discuss the possible effects on the efficacy of vaccinations, after having shortly revised the basics of the immunology of vaccinations themselves.

For the purpose of this review, articles were identified using the PubMed database through a comprehensive search conducted by combining key terms such as “pollution”, “vaccines”, “inflammation”. English language articles were screened for relevance.

In Table 1, a summary of the different forms of pollution, possible impact on human health, and main references is shown.

## 2. Consequences of Pollution on the Immune System

For the first time ever, in 2020 air pollution was recognized as a cause of death: the victim, a young girl suffering from asthma, died after a particularly severe asthma attack after being exposed to air pollutants [22]. The decision highlighted the importance of pollution on human health, not only through environmental changes but also through direct effects.

**Figure 1:**
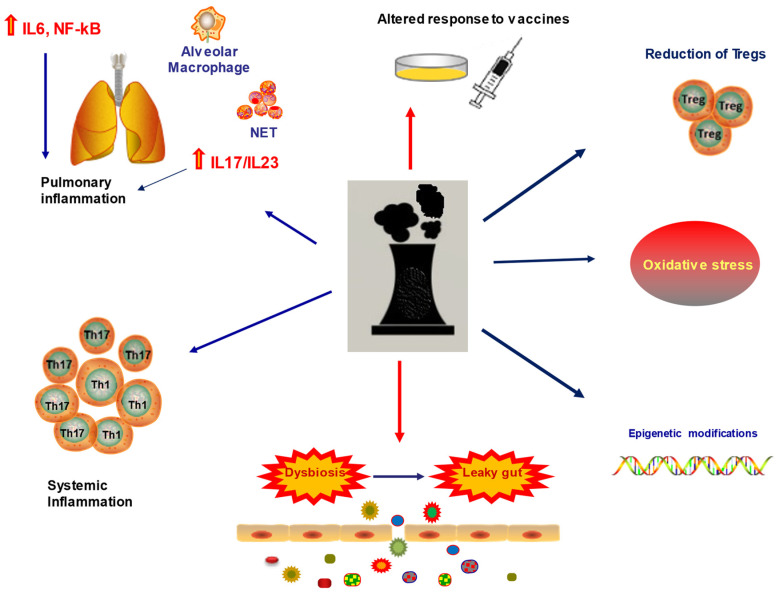
Impact of pollution on human health. Pollution impacts human health on many levels: on the immune system, enhancing pulmonary, intestinal, and systemic inflammation. Pollution can increase the risk of developing cancer through epigenetic modifications and oxidative stress. Pollutants can directly affect the response to vaccines. Abbreviations: NET: neutrophils extracellular traps, IL: interleukin; Tregs: T regulatory cells.

Pollution impacts human health on many levels (Figure 1), but the public tends to underestimate its consequences, which impact politics and behaviors [23].

Pollutants can stimulate and interact with the immune system through different paths, one of them is stimulating inflammation: Chen et al. have studied the effects of particulate matter on elderly persons, both with and without chronic obstructive pulmonary disease. Interestingly, the patients who were most affected were those who did not suffer from other inflammatory pulmonary diseases [24].

Air pollution appears to be capable of determining widespread inflammatory responses, which can, in turn, determine a wide variety of diseases, through chronic inflammation [25,26,27].

Not only particulate matter has the capacity to trigger inflammation: something similar can be observed with pollutants more typical of water and land, such as microplastics; studies on animals show that ingestion of this material can cause bowel inflammation and microbiota disruption, which translates to more widespread disorders [28]. Even though the thought of finding petrol in everyday drinking water and food is not appealing, this is also another kind of pollutant that has important consequences on human health, particularly in terms of inflammation [29,30,31].

Exposure to particulate matter and microplastics activates different inflammatory pathways. Oxidative stress is probably a key mechanism: generating reactive oxygen species (ROS), many different inflammatory pathways are activated, depending on the damaged tissue. While at low doses, ROS actually exert a positive function, when at high levels, they can damage different cell components, leading to an inflammatory response [32].

In the lung, which is one of the most directly targeted tissues, interleukin (IL)-1, IL-6 [33], and tumor necrosis factor (TNF) are secreted by T-lymphocytes upon damage. The activation of this pathway also determines the production of C-reactive protein (CRP) and serum amyloid A (SAA), while also determining a more localized inflammation, through polymorphonuclear leukocytes [34]. It is worth noting that the relationship between pollutants and oxidative stress is not directly proportional but also depends on its components, particularly metals and soluble substances [35].

IL-1β and TNF-α are key players also in cerebral inflammation following exposure to pollution: when exposed to air pollution, the microglia starts producing higher quantities of inflammatory cytokines, which can chronically lead to disequilibrium in the central nervous system, and to the development of neurodegenerative disorders [36], given that pollution exposure leads not only to a change in cytokine profile but also alters the expression of other key mediators for vascular and cellular health, such as inducible nitric oxide synthase (iNOS) [37]. Microglia plays a very important role in the inflammatory response that takes place when the central nervous system is exposed to pollution. For instance, a study con-ducted in Mexico City demonstrated that exposure to diesel exhaust particles (DEP) in animals drives neuroinflammation, activating the Mac1-NOX2 (NADPH oxidase) pathway [38].

Another activated inflammatory pathway is the Aryl hydrocarbon receptor (AHR) pathway. The activation of AHR leads to a series of consequences, particularly involving Th17 lymphocyte differentiation. Indeed, regulation of Th17 by IL-2 is reduced, and so are Signal Transducer and Activator of Transcription (STAT)-1 and -5. At the same time, immunosuppression is also stimulated through the activation of c-MAF or STAT-3 [39].

Inflammation is also driven by other mechanisms: in the case of the gut, fibroblasts and epithelial cells are the activators of the immune system after exposure to pollution, through direct production of inflammatory cytokines (particularly TGF-β, IL-10, IL-17), but also changing the expression of toll-like receptors (TLRs) and surface proteins [40]. Petrol, which interestingly is a key pollutant for the gut, is also known to activate directly IL-17, TNF, and other cytokines, similarly to pesticides [41].

The inflammatory response to environmental pollutants is also driven by more complex mechanisms, such as micro-RNAs, which can involve different genes and processes [42] and DNA alterations. In particular, the exposure to particulate matter leads to anti-ICAM, IL-1β, and TNF-α activation, following DNA damage [43]

In some cases, the inflammatory effects of different pollutants can act in a synergic fashion: for instance, ozone can react with carbon black, producing a fulvic acid-like substance, which has a high inflammatory potential [44].

The impact of pollution on the immune system goes beyond inflammation, as some substances can directly act on it: Perfluorinated Alkyl Substances (PFASs) directly influence Th1 and Th2 pathways, also leading to higher immunoglobulin (Ig)-E levels [45]. PFASs seem to impact more specifically cytokine production by Th1 and Th2 lymphocytes, stimulating the latter (IL-4 and IL-5), while inhibiting the former (interferon (IFN)-γ, IL-2) [46]. PFASs can determine their effects in particularly delicate populations, such as newborn babies; as seen in a Canadian study, exposure to PFASs and bisphenol A can lead to alterations in the levels of IgE, IL33, and thymic stromal lymphopoietin (TSLP) [47]. Nuclear factor-κB (NFκB)–inhibitor of κB kinase, c-Jun N-terminal kinase and activator protein-1 (JNK-AP1), and inflammasomes are other key immune components in determining the interactions between the body and PFASs [48]. In some cases, the synergic effect takes place between pollutants and pathogens, which can determine direct cellular damage, resulting in altered immune responses. During the current COVID-19 pandemic, this mechanism has been used to explain the higher prevalence of the disease in more polluted areas [49].

Overall, it appears clear that the immune system is the first to react to pollutants, but it is not left unshattered by the interaction. Below we will discuss the possible effects of the interaction between the immune system and pollution, focusing on three main areas, also linked to vaccination response, summarized in Table 2.

### 2.1. Pollution and Autoimmune Disease

Inflammation is an important trigger in autoimmune conditions; it is no surprise that pollutants can play a key role in initiating these disorders: Zhao et al. have studied the effects of air pollution, specifically on the immune system [50]. The effects of air pollution in terms of inflammation can be determined by many different components of the pollution itself, which includes both gasses and particulate matter. Some metals present in the particulate can act as adjuvants, enhancing inflammation. An autoimmune/inflammatory syndrome induced by adjuvants (ASIA) is a described autoimmune condition, triggered by adjuvants [51]. Interestingly, ASIA was first described in relation to vaccination [52]. Overall, it appears that pollution may promote the development of autoimmune disorders through chronic inflammation, which can then also impact the efficacy of vaccinations.

In particular, [53] oxidative stress triggers various immune pathways. Mitogen-activated protein kinase (MAPK) and nuclear factor kappaB (NF-κB) are particularly relevant, activating uncontrolled inflammatory reactions, which are important risk factors in the development, for instance, of multiple sclerosis [54].

Oxidative stress in the airways also increases after exposure to air pollution, activating NF-kB and stimulating the production of Th1 lymphocytes. Also, alveolar macrophages and airway epithelial cells start expressing pro-inflammatory cytokines, stimulating dendritic cells to migrate toward local lymph nodes, causing cell necrosis and apoptosis. Neutrophil extracellular traps (NETs) are released, and IL-17 and IL-23 levels increase. All these reactions induce an inflamed niche [55].

Oxidation can also impact processes of methylation of genes, particularly of those involved in inflammatory pathways [56]. While on the one hand systemic inflammation, epigenetic modifications, and oxidative stress are key factors, air pollution seems to be able to activate the AHR, which activates the Th17 subset of T-lymphocytes, while down-regulating T-regulatory cells (Tregs). Overall, there appears to be an imbalance in the Th1/Th17 subsets [50]. Even though the above-described mechanisms can apply to any autoimmune disease, there appears to be a stronger predisposition to develop certain diseases, rather than others: rheumatoid arthritis (RA), systemic lupus erythematosus, and type 2 diabetes [57]. In these diseases, different mechanisms might be playing a role; oxidative stress is one of them. In RA, ROS are commonly found at the site of the disease and are a driving force for the disease [32].

PFASs also play an important role in triggering autoimmune diseases. The thyroid is particularly sensitive to their effects: a higher exposure to them increases the risk of developing Hashimoto’s disease, as observed in epidemiologic studies in the South of Italy [58]. In those same areas, also multiple sclerosis and other immune-mediated disorders are on the rise [59].

It has also been observed that pollution may significantly enhance the allergic power of pollens in susceptible individuals, which has dire consequences in terms of quality of life, but also in terms of chronic inflammation [53]. It is still debated whether vaccinations are safe and effective in persons with autoimmune disorders: even though the most recent guidelines did not highlight any increased risk and only a slight reduction in effectiveness of some vaccines, the results are still not considered conclusive [60].

### 2.2. Pollution and Cancer

The link between environmental factors and the development of cancer is well known; in polluted environments, the link is even stronger. The so-called “Land of Fires” in Campania, site of dumping of hazardous material, is a perfect example: people living in this area have a very high risk of developing many different forms of cancer, ranging from breast, to prostate, to lung cancer and even child leukemia [61]. Even in areas where the situation is not as dramatic, the consequences of water and air pollution are still staggering: not only lung cancer is linked to air pollution [62], but also urological forms, particularly bladder cancer [63], breast and prostate [64,65]. Metals in water increase the overall risk of developing cancer [66], and populations living in industrial areas face an enormous disease burden, which is mostly caused by pollution [67,68].

The mechanisms through which pollution (not including in our discussion nuclear waste) can increase the risk of developing cancer are not completely clear, but it has been hypothesized that they are similar to the ones described in the context of autoimmune diseases [69]. One of the mechanisms that has been described is DNA methylation: it appears that some pollutants are capable of determining alterations in DNA methylation, both in loss and excess [70].

Oxidative stress is also linked to the development of cancer, through the promotion of inflammation and activation of the immune system [71]; both the adaptive and the innate components are activated. There are various consequences: on the one hand, the immune system struggles to fight infections, on the other, downregulation of Tregs has a negative impact on the capacity to recognize self. Both these situations can determine a chronic low-grade inflammation which can have negative consequences and favor the development of neoplastic conditions [72]. Interestingly, oxidative stress may also play a role in cancer outcomes [32],

PFASs have also been under scrutiny for their potential carcinogenic effect on people, given they can directly also modulate the endocrine system, leading to dangerous hormonal imbalances. Even though some evidence has been collected on the increased risk of developing particularly kidney and testicular cancers, other studies have found conflicting results as for prostate, bladder, pancreatic, or liver cancer. In the end, the International Agency for Research on Cancer (IARC) has classified PFASs as “possibly carcinogenic” for humans [73]. Yet, some studies suggest that the exposure to air pollution may improve outcomes in some types of cancers, for instance, lung cancer [74].

In this case, again, the interaction between the immune system and pollution is guided by inflammation. Also, chronic inflammation can impair vaccination response in cancer patients, similarly to autoimmune disease, which is particularly dangerous given that this part of the population is most at risk of developing fatal consequences if infected with diseases such as influenza [75,76].

### 2.3. Pollution and Gut Microbiota Composition

Air and water pollution can impact gut microbiota homeostasis in different ways. It has been observed that exposure to air pollution directly affects microbiota composition, not only in mice models [77], but also in human ones: particulate matter has the capacity to determine direct inflammation in the gut, favoring dysbiosis, a higher prevalence of *Firmicutes* and *Bacteroides caecimuris*, and a lower bacterial variability [78,79,80]. At the same time, air pollutants also help gut permeability, determining a condition called “leaky-gut syndrome” [72]. All these conditions together further enhance the inflammatory effects of air pollution, determining its systemic consequences.

The most direct forms of pollution for the gut, though, are water and land pollution, given that they both influence what we eat and drink, thus directly interacting with our gut.

Water pollution has an impact on gut homeostasis, sometimes in more direct ways: even in drinking water the levels of antibiotics are high, and this can directly impact gut microbiota composition [10]. Other types of water pollution can also have important effects; for instance, the presence of heavy metals (sodium arsenite and cadmium chloride, in particular) has been linked to a lack of diversity in microbiota composition, which might in part drive the development of diseases such as type 2 diabetes [81]. The presence of heavy metals in water can also be associated with the presence of antibiotic-resistant microbial communities, with even more serious consequences: it has been observed that the presence of zinc is associated with resistance to oxacillin, cefotaxime, trimethoprim [82]. Microplastics are another dangerous form of water and land pollution: their effects are mechanical (malnutrition, inflammation), chemical (endocrine disruptors), biological (pathogens, dysbiosis). The last effect is particularly disruptive for gut homeostasis [83,84]. The consequences of these effects are not yet visible, but the rise in the levels of microplastics globally is a cause of concern [85].

PFASs can also directly impact gut microbiota: a decrease in the proportion of *Bacteroidetes* and an increased proportion of phylum CKC4 has been observed in animals exposed to bisphenol A, while in general there are alterations in the delicate equilibrium of microbiota, expressed by the *Bacteroidetes*/*Firmicutes*. Bacilli (e.g., *Lactobacillus*), *Bacteroidetes*, *Proteobacteria*, and *Actinobacteria* are all impacted by PFASs [86].

An interesting aspect that is not yet completely understood is the bidirectional interaction between pollutants and microbiota: for instance, polycyclic aromatic hydrocarbons and heavy metals can undergo bioremediation by different bacteria and fungi [87]. Microbiota acts as an immunomodulator and is also involved in the response our organism gives to vaccination: different types of bacteria inhibited by PFASs, for instance, are also linked to a better immune response to vaccination and overall longevity [88].

## 3. Vaccination and Immune System

Vaccination is a safe and effective measure against a great number of diseases and has been deemed responsible for cutting down child mortality [89]. Yet, many people are still skeptical about their effectiveness and suggest that they may cause different diseases, including autism [90]. The underlying reason is probably the way vaccinations are administered, given that the general public often sees them as the inoculation of the diseases they should prevent. The fact that they are administered, in particular, to a delicate population, such as children, further explains the suspicion they can raise [91].

In the past, the vaccination process was indeed based on the idea to administer low or weakened doses of the pathogen, to avoid severe infections, as done by Jenner in the case of smallpox [92] and then by Pasteur for rabies [93]. Over time vaccine technology has evolved and is now more refined, safe, and effective [94,95].

A recent example of this is the design of the vaccine against Sars-CoV-2, the pathogen responsible for COVID-19 disease [96]. While different methods have been used to try to produce vaccines, the most interesting is the one based on mRNA technology, in which the host interacts with the inoculated mRNA and produces antibodies without having to be exposed to even the smallest viral particle. While some express concern for those suffering from autoimmune conditions, this type of vaccination is safe and effective [97].

A vaccination is effective if it determines an adequate immune response, obviously, an adequate immune response also depends on the conditions of the vaccinated individual. Age, sex, and immune status are all linked to different responses to vaccinations [88,98]. It has even been suggested that baseline immunity should be studied in order to design personalized vaccination strategies, to maximize the immune responses of each individual [99].

Environmental factors also play a significant role. Firstly, in developing countries, especially in rural areas, malnutrition is the more frequent pathological condition that severely affects functions of T and B cells with consequent impairment of antibody production; it may decrease complement and phagocyte activity [100]. These impaired functions have a key role not only in favoring infectious diseases but also in the impairment of vaccine response. Corrections of low levels of vitamins, iron deficiency, and protein intake could improve the immune-regulatory system [101]. Moreover, it has well been demonstrated that the area of residence with the associated microbial environment may play a role in the development of immune responses. The ‘hygiene hypothesis’ links a minor exposure to infections during childhood to a minor regulation of Th1, Th2, and Th17 inflammatory responses. However, some authors have observed that children living in urban areas of the tropics had significantly increased levels of IL-10 [102]. Moreover, it has been observed that children living in the countryside have an increased response to vaccinations when compared to children living in cities. The difference is based on differences in the activated immune pathway: when living in rural areas vaccination activates a Th1-skewed response (IL-5 production) while in an urban context it activates a Th2-skewed response (IFN-γ production) [103].

The importance of the Th1 immune response in enhancing vaccination efficacy is proven by the fact that in populations who tend to have defects in this immune response, vaccinations are not as effective.

An example of this is the lower immune response that elderly people exhibit to vaccination [104]: indeed, an imbalance towards Th17 pathway expression, which penalizes Th1, is a key factor lowering the efficacy of vaccines in this group [105].

Other immune pathways are involved in the vaccination process, depending on the pathogen. Cytotoxic lymphocytes CD8+, for instance, need to be activated for pathogens entering cells, and cancer [106], while delayed hypersensitivity, involving CD4+ lymphocytes and monocytes, is fundamental in tuberculosis, leprosy, syphilis, and fungal infections [107].

Another interesting aspect is that, while the immune system impacts the way we react to vaccination, vaccinations also have the potential to shape our immunity. Studies have concentrated on different populations, particularly those with a higher risk of not being immune-competent. Neonates, for instance, are at a particular risk of infections, which may carry serious consequences. On the one hand, there are specific vaccines against specific pathogens, on the other, it is not possible to vaccinate against all potential pathogens. A possible alternative strategy is to administer immune boosting vaccinations, which should stimulate immune response overall [108]. Immune boosting vaccination strategies include homologous and heterologous prime boosting approaches, which have been tested in pre- and clinical trials [109].

Vaccinations boosting immunity have also been a staple in other populations, particularly cancer patients. In this case, vaccinations are designed to activate immunity against cancer-specific antigens or to stimulate the patient’s immune system to attack the cancerous cells [110].

While some interactions between the immune system and vaccinations are desirable, some others are not: for instance, in some persons, vaccination has been identified as a trigger for autoimmune disorders, and there still is not a consensus around vaccinations in people who are suffering from these diseases. The underlying mechanisms in this interaction are complicated: adjuvants, for instance, can trigger immune activation, similarly to what happens with some pollutants; antigens can also trigger immune activation which, combined with molecular mimicry, can determine autoimmune disorders; finally, other components of the vaccination, can determine allergic reactions, activating, once again, the immune system [111]. Interestingly, inadequate immune activation of the immune system caused by pollutants has also been observed and will be discussed below.

The wide involvement of the immune system in vaccine response justifies the importance of the environment in modulating responses to vaccination [112]. Also, a chronic inflammatory status seems to reduce the organism’s capacity to correctly respond to vaccinations, as it involves almost all immune pathways [113]. The impact of pollution on chronic low-grade inflammation could in part explain its impact on vaccinations.

## 4. Possible Consequences of Pollution on the Effectiveness of Vaccines

Response to vaccination depends on the conditions of the host, which also depend on the surrounding environment [114]. The interactions between the immune system and different types of pollutants, described above, can impact the response to vaccination, but in some cases, direct interactions between some forms of pollution and vaccination response have been reported.

Pesticides, for instance, are fundamental for the control of diseases linked to animal vectors, as in the case of malaria. Bendiocarb is a pesticide that is transferred to the fetus during pregnancy, and it has been shown to have immunological effects on fetal immune cells, with fewer Tregs and increased levels of inflammatory cytokines, with a consequent increase of inflammatory responses. Surprisingly, this dangerous in utero exposure has been shown to enhance measles vaccine responsiveness [115].

On the other hand, the exposure to phthalic acid esters (PAEs) used as plasticizers [116] has been associated with a decrease in hepatitis B antibody concentrations in children [117]. Moreover, the exposure to polycyclic aromatic hydrocarbons (PAHs), which are environmental contaminants, could affect Hepatitis B vaccine-induced antibodies concentrations [118].

Furthermore, it has been demonstrated that the anti-hepatitis A virus immunoglobulins (Ig)-M present different titers in different seasons. The IgM increased from January to April and decreased from July onwards. This difference seems to be linked to groundwater pollution during the spring drought [119].

PFASs (including perfluorooctanoic acid, PFOA, and perfluoro octane sulfonate, PFOS) are ubiquitous chemical substances, and exposure to them has been associated to a decrease in the humoral immune response to tetanus and diphtheria vaccines in children [120]. Similarly, higher maternal PFAS levels are linked to low rubella vaccine antibodies in early childhood [121].

Moreover, a recent report has shown that high levels of PFOA are associated with low rubella vaccine antibodies in adult men [122]. PFOA exposure also causes a lower influenza vaccine response in adult people [123].

In a recent study, the levels of PFOA have been shown to be associated with lower levels of IFNɣ in ex-vivo cells after stimulation with tetanus and diphtheria toxoid and to a lower production of anti-Haemophilus influenza type b, anti-tetanus, and anti-diphtheria antibodies [124].

Air pollutants, such as hydroquinone (HQ), a tobacco particulate compound, also have an impact on the immune response to vaccination. HQ, for instance, modifies the morphology of spleen follicles and increases the lymph nodes in mice. Even though these reactions do not seem to directly modify titers of influenza vaccine-specific IgG [125], it has been recently demonstrated that secondhand smoke is linked to a strong impairment of immune response to specific vaccines in murine models [126].

Yet, the relation between vaccination and pollution is not monodirectional: a cross-sectional study on 6740 Chinese children aged 7–14 years, for instance, has shown that influenza vaccines could reduce the negative effects of air pollution on lung function, particularly in girls more than in boys [127]. Influenza may also protect against negative effects of pollution elderly persons suffering from acute coronary syndrome (ACS): data on 1835 elderly patients suffering from ACS was collected and consecutive influenza vaccination offered protection against detrimental environmental factors [128].

In Table 3, a short summary of the main pollutants associated with decreased immune response is shown.

Overall, the reported results highlight the existence of a complex crosstalk between vaccinations and pollution. The interesting aspect is that, even though there still are many question marks on what will happen in the future, it is still possible to identify problematic areas in vaccinations, consequent to environmental population.

The efficacy of vaccines depends on the integrity of the immune system. The impact of pollution on immunity is documented, thus it is reasonable to assume that pollution might negatively impact the efficacy of vaccinations. While it is encouraging that vaccinations might help to prevent some of the side-effects of pollution, it is worth noting that those who are exposed to the highest levels of it are those living in the poorest areas of the world and are also the ones who have the lowest access to vaccination programs [129,130]. This is particularly true for children and older people, who are also the ones who would benefit the most from prom vaccination programs. Also, pollution triggers immune responses similar to those seen in autoimmune diseases [39]. Response to vaccinations in patients suffering from these conditions has been analyzed and seems not to be as strong as in the general population [131] and can sometimes even worsen the underlying condition [132]. While there is no evidence at the moment that the interaction between vaccinations and pollution could lead to autoimmune disorders, it is worth noting that the impact of chronic inflammation on vaccinations is negative in terms of efficacy, as seen in inflammaging or in other similar chronic inflammatory conditions [88]. On the other hand, chronic inflammation can trigger the development of autoimmune disorders [133,134,135] and so can vaccinations, in particularly at-risk categories [136]. Thus, it is not possible to rule out a synergic effect in people who are predisposed. At the same time, pollution-associated chronic inflammation might trigger autoimmune responses, increasing the effective population at risk.

It is important to remember that the above-listed consequences of the interaction between vaccination and immunity are a social issue as well, besides being a medical one, given their impact on lower-income countries rather than on the higher-income ones [137,138].

Yet, this does not mean that higher-income countries should not be concerned, given that the world is not made of watertight compartments.

Besides the obvious consideration that we need to rethink our lifestyle to reduce our impact on the environment, it also is important to further study the consequences of pollution on vaccinations, given the possible consequences on future vaccination programs.

Indeed, until we do not fully understand the interactions between vaccinations and pollution, we will not be equipped to face the challenges ahead of us, with dangerous consequences for global health.

## 5. Conclusions

The importance of the environment on human health has always been well-known, but the impact of pollution is yet to be completely understood, even though it threatens the most vulnerable parts of our population. The events that took place in Flint, Michigan, and in the “Land of the Fires” in Campania are an obvious demonstration that the environmental crisis is a real risk and not something only scientists worry about.

Yet, the implications on human health are far wider than only the direct consequence of pollution, and our immune system is directly under attack, determining, for instance, chronic inflammation and overall immune dysregulation. The consequences of these events are diverse, ranging from autoimmune disorders to cancer. Vaccinations also have an important effect on immunity; thus, it is likely that a crosstalk between vaccines and pollutants may have important consequences on human health. On the one hand, the interaction between vaccinations and pollution may protect against the consequences of the latter, yet it could also lead to further immune dysregulation. Another risk is the possible loss of efficacy of vaccinations for those living in a polluted environment, which could push further the no-vax movement.

While further studies are needed to confirm the interactions between vaccinations and pollution and their consequences, it is likely that the most affected populations will also be the most fragile ones, thus transforming the issue from a mere medical problem to a social one as well, also because low-income countries are the ones most at-risk.

## Figures and Tables

**Table 1:** Types of pollution and possible impact on human health.

Type of Pollution	Description	Health Consequences	References
Air	Presence of gases, biological molecules, and particulate matter in the atmosphere.	Air pollution significantly impacts health. Ozone and particulate matter are two of the most important components and determine a high number of hospitalizations yearly.	[11]
Water	Contamination of water bodies with different kinds of pollutants.	Sewerage, pesticide, and industrial water pollution affect a large part of the population, leading to diarrhea, bowel inflammation and other.	[12]
Soil contamination	Degradation of land and soil, caused by pollutant contamination.	Industrial and pesticide contamination of the soil have been linked to adverse health outcomes, particularly if in agricultural sites.	[13]
Noise	Unpleasant and disturbing sounds that disturb the equilibrium of that environment.	Noise pollution has been linked to hearing loss and alterations. Other pathologies, such as psychiatric and cardiovascular disorders have also been linked to this form of pollution	[14,15]
Plastic	Plastic accumulation in the environment.	Plastic is dangerous for human health on different levels, in particular, in its micro- and nanoparticles, which can be ingested and determine direct effects on the organism.	[16]
Radioactive contamination	Presence of radioactive material.	Radioactive contamination of the environment can have immediate and long-term consequences. In particular, cancer and cardiovascular disorders are common among those chronically exposed to radiation	[17]
Light	Anthropogenic light disrupting nocturnal environment.	Light pollution has been linked to disruption of circadian rhythms with various consequences on hormonal cycles, which can in turn determine stress related conditions, cardiovascular and endocrine disorders.	[5,6,18]
Thermal	Induced change in water temperatures.	Thermal pollution is often a consequence of industrial efforts. It can directly impact the water, altering the species living in it, and promoting water pollution, and also impact the quality of air, raising temperatures, further enhancing the negative effects of air pollution.	[19]
Visual	Presence of barriers disrupting the surrounding environment.	Visual pollution is usually associated to the construction of buildings and infrastructures, where there were none before. While it is difficult to state whether it has a direct impact on human health, it is associated to other forms of pollution.	[20]
Littering	Human waste not properly disposed of.	The presence of litter in the environment is, in itself, another form of pollution. Usually, it is caused mostly by the presence of microplastics in the litter, which can then contaminate the soil and water.	[21]

**Table 2:** Effects of pollution on health and vaccination.

Diseases	Effect of Pollution	References
Autoimmune diseases	Triggered by different types of pollutants, creating low-grade chronic inflammation, possibly impairing response to vaccination.	[60]
Cancer	Different pollutants are involved in the development of different forms of cancer, dysregulating immunity and triggering inflammation.	[75,76]
Gut microbiota	Microbiota acts as an immunomodulator and is also involved in the response that our organism gives to vaccination: different types of bacteria inhibited by PFASs, for instance, are also linked to better immune response to vaccination and overall longevity	[88]

**Table 3:** Pollutants and vaccines.

Pollutant	Effect on Immunity and Vaccination	References
Bendiocarb	Reduced Tregs, increased inflammation; better response to measles vaccination.	[115]
PAE	Reduction of HBV-antibody titers in children.	[117]
PAH	Reduced HBV vaccine response	[118]
PFAS	In utero exposures are linked to low rubella vaccine antibodies in early childhood and decreased humoral immune response to tetanus and diphtheria vaccines in children.	[120,121]
PFOA	Low rubella antibody levels in adults; reduced response to influenza vaccination; lower production of anti-Haemophilus influenza type b, anti-tetanus, and anti-diphtheria antibodies.	[122,123,124]
HQ	Impairment of immune response to vaccination in murine models.	[126]

## Data Availability

Not applicable.
